# A novel three-base duplication, E243dup, of *GFAP* identified in a patient with Alexander disease

**DOI:** 10.1038/hgv.2017.28

**Published:** 2017-07-06

**Authors:** Rei Yasuda, Tomokatsu Yoshida, Ikuko Mizuta, Masanori Nakagawa, Toshiki Mizuno

**Affiliations:** 1 Department of Neurology, Graduate School of Medical Science, Kyoto Prefectural University of Medicine, Kyoto, Japan; 2 Department of Neurology, North Medical Center, Kyoto Prefectural University of Medicine, Kyoto, Japan

## Abstract

Alexander disease (AxD) is a rare hereditary neurodegenerative disorder caused by glial fibrillary acidic protein (*GFAP*) gene mutations, most of which are missense mutations. We present an AxD case with a novel *de novo* three-base duplication mutation in *GFAP* resulting in E243dup.

Alexander disease (AxD, OMIM #203450) is a rare autosomal dominant neurodegenerative disorder that is pathologically characterized by cytoplasmic inclusions called Rosenthal fibers in astrocytes. Since mutations of glial fibrillary acidic protein* *(*GFAP*) were identified as the cause of AxD in 2001,^1^ genetic testing of *GFAP* has become a powerful tool for the diagnosis of AxD. Here, we report an AxD patient with a novel *GFAP* three-base duplication mutation.

A 27-year-old woman with blurred vision was referred to our department because of abnormalities noted on brain magnetic resonance imaging (MRI). She had been healthy until 5 years earlier, when slowly progressive gait disturbance and dysphagia developed. She had no family history of neurological disorders. On neurological examination, saccadic eye movement, gaze nystagmus, slurred speech, hypotonus of the extremities, exaggerated muscle stretch reflexes of the right Achilles tendon, and ataxia of the upper extremities and body trunk were observed. The score of the Japanese version of Montreal cognitive assessment (MOCA-J) was 23/30, indicating mild cognitive impairment (normal cutoff score >26). Brain MRI showed atrophy of the medulla oblongata, as well as T2 hyperintensity of the ventral medulla oblongata, dentate nuclei of the cerebellum, cerebellar peduncles, and frontal dominant periventricular white matter (Figure 1a,Figure 1b,Figure 1c,Figure 1d). On the basis of these clinical features and MRI findings, we considered a diagnosis of AxD and performed genetic testing of *GFAP*. A novel heterozygous three-base duplication mutation (c.726_728dupAGG) in exon 4 of *GFAP* was identified (Figure 1e). This mutation resulted in duplication of the glutamate residue (p.E243dup). The mutation was not found in either of her parents.

On the basis of its clinical manifestations and imaging characteristics, AxD is classified as cerebral AxD (type 1), bulbospinal AxD (type 2), and the intermediate form (type 3).^2^ Type 1 AxD is characterized by a mainly infantile onset, convulsions, macrocephaly, psychomotor developmental delay and frontal dominant white matter abnormalities on brain MRI. Type 2 AxD is characterized by a mainly adult onset, muscle weakness, spasticity and bulbar symptoms, reflecting an abnormal signal or atrophy of the medulla oblongata and/or cervical cord on MRI. Some type 2 AxD patients show cerebellar ataxia with signal abnormalities and/or atrophy of the cerebellum on MRI. According to these characteristics, our case can be classified as type 3 AxD. In this case, the most critical factor to suspect AxD was the characteristic MRI findings. The findings were frontal dominant white matter lesions and abnormal signals in the medulla oblongata, which are called the ‘eye spot’ sign in typical elderly onset cases.^3^

From genetic testing, a novel mutation, E243dup, was identified in *GFAP*. We concluded that this mutation was pathogenic for the following reasons. First, it was predicted to be ‘deleterious’ by Protein Variation Effect Analyzer (PROVEAN, http://provean.jcvi.org/). Second, the GFAP coil 2A domain is highly conserved among species, both in length and amino acid sequence (Figure 1f). Third, this mutation was a *de novo* mutation because it was not identified in her healthy parents. Fourth, the mutation is not present in nucleotide variation databases, including Exome Aggregation Consortium (ExAC, http://exac.broadinstitute.org) and Human Genetic Variation Database (HGVD, http://www.genome.med.kyoto-u.ac.jp/SnpDB). Finally, mutations that alter the amino acids adjacent to E243, Y242D^4^ and A244V^5^ have already been reported in AxD.

We previously reported that the mutations in adult cases are distributed widely in *GFAP*, whereas most of the mutations in infantile cases are located in R79, R88 or R239.^2^ GFAP consists of head, rod and tail domains. The rod domain is highly conserved and includes four alpha-helical subdomains (coil 1A, 1B, 2A and 2B).^1^ The E243dup mutation we identified is a three-base insertion located in coil 2A (Figure 1f). Of the >100 *GFAP* mutations reported in AxD, the majority are missense, and a few are in-frame insertions/deletions. The previously reported in-frame insertions/deletions were located in the alpha-helical subdomains of the rod and were all associated with infantile- or juvenile-onset AxD, except for the case with R124_L125insQLR (Figure 1f). Our case is the second report of adult-onset AxD with an in-frame mutation.

Here, we report a novel three-base insertion, the E243dup mutation in *GFAP*, causing AxD. The characteristic MRI findings, including frontal dominant white matter lesions and abnormal signals in the medulla oblongata, prompted us to perform genetic testing of *GFAP*. Considering the markedly low prevalence and wide-ranging mutation variations of AxD, reporting novel mutations with detailed clinical information is helpful for the diagnosis of AxD in suspected cases.

## Accession codes

Accession number: LC214884 (available at http://www.ddbj.nig.ac.jp/).

## Figures and Tables

**Figure 1:**
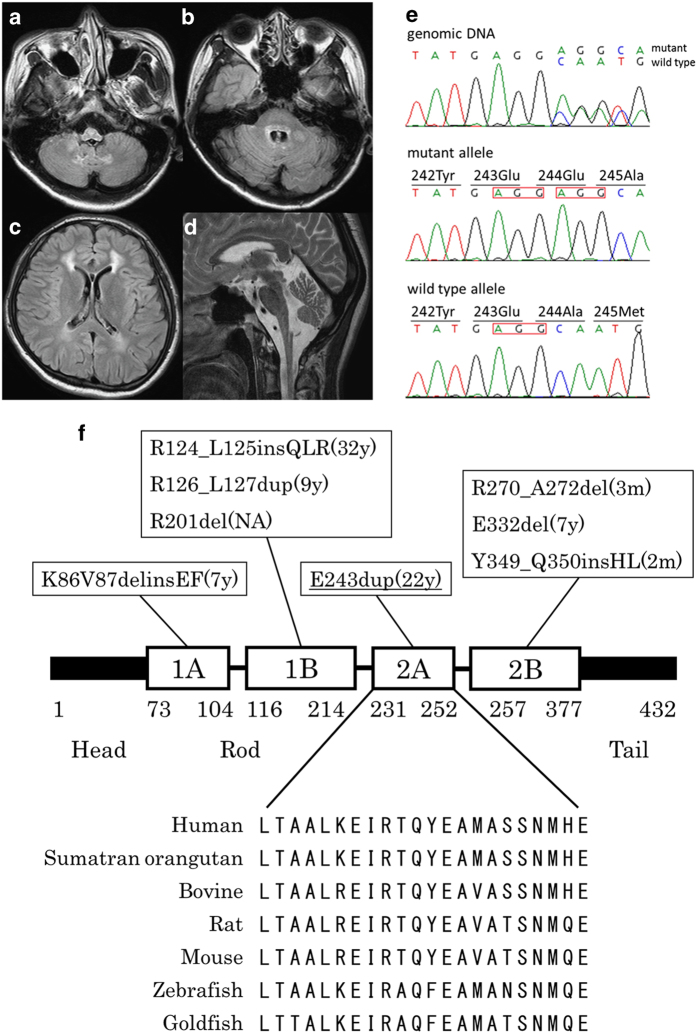
Brain MRI and genetic testing. (**a**–**d**) Axial fluid-attenuated inversion recovery (**a**–**c**) and sagittal T2-weighted (**d**) brain MR images showed atrophy and abnormal signals of the medulla oblongata (**a**,**d**), abnormal hyperintensities in the dentate nuclei and middle cerebellar peduncles (**b**), and frontal dominant periventricular white matter lesions (**c**). (**e**) Chromatograms of genomic DNA (upper), the subcloned mutant allele (middle), and the wild-type allele (lower). There was a heterozygous c.726_728dupAGG (red box) mutation in *GFAP* gene exon 4 that resulted in E243dup. (**f**) Summary of the GFAP protein structure and the location of insertion/deletion mutations (references cited in http://www.waisman.wisc.edu/alexander-disease). The alignment of the amino acid sequence of the coil 2A domain among species is also shown. The onset ages of the reported cases are in parentheses. Our case is underlined. The amino acid number is shown. m, months; MRI, magnetic resonance imaging; NA, not available; y, years.
